# Supplementary material for: Translation and interpreting teachers’ perceptions of dilemma and needs in their professional development
Source: PLoS One. 2024 Aug 9;19(8):e0289269. doi: 10.1371/journal.pone.0289269 (PMC11315291; doi:10.1371/journal.pone.0289269)
Supplement: S1 File — (DOCX) [file pone.0289269.s002.docx]

**Translation and interpreting teachers’ perceptions of dilemma and needs in their professional development**

**Semi-Structured Interview Guide**

1. Why did you choose to be a teacher for MTI program?
2. How do you view the current MTI education?
3. As an MTI teacher, could you please elaborate on your current situation in terms of teaching practice, academic research, translation or interpreting practice, professional title evaluation and promotion, and teacher learning?
4. What difficulties have you encountered an MTI teacher?
5. What qualities or competencies do you think an excellent MTI teacher should possess?
6. What factors affect your professional development as an MTI teacher?
7. As an MTI teacher, how do you balance teaching, research, and translation or interpreting practice?
8. What are the needs for your professional development?
