# Supplementary material for: Translation and interpreting teachers’ perceptions of dilemma and needs in their professional development
Source: PLoS One. 2024 Aug 9;19(8):e0289269. doi: 10.1371/journal.pone.0289269 (PMC11315291; doi:10.1371/journal.pone.0289269)
Supplement: Attachment — Submitted filename: Response to Reviewers.pdf [file pone.0289269.s003.pdf]

Yi Liu and Jianyu Liu
Liaoning University
Shenyang, P. R. China
liuyi@lnu.edu.cn, ljy_0923@163.com

20 June, 2023

Dear Editors and Reviewers,

We are grateful for the opportunity to improve this manuscript to *PLOS ONE*, and we do appreciate the insightful comments given by the editors and reviewers. We have carefully revised our manuscript according to the comments as indicated below.

Reviewer 1:

- ***Comment (1) and (2):***

(1) The "Introduction" part is too lengthy.

(2) There should be a separate "Literature Review" Part. The paper should exhibit an adequate understanding of the relevant literature in the field. There is a need to update recent literature up to 2023.

☐ **Response:**

Thank you very much for your constructive suggestions. In the revised manuscript, we reorganized the "Introduction" part (Lines 34-62) and added a separate "Literature Review" part (Lines 63-99). In the "Literature Review" part, we reviewed the literature of the relevant research about translation and interpreting teachers and updated recent literature up to 2023. The changes to these two parts are highlighted in blue in the revised version.

Reviewer 2:

- ***Comments (1) and (2):***

(1) Inclusion of "Limitation" of this study would add value and is suggested accordingly.

(2) It is suggested that the author should include "Research Objective" section and the "Research Questions" be moved out from the "Methodology" section and placed ahead of the "Methodology".

☐ **Response:**

Thank you very much for your positive feedback with constructive suggestions for this manuscript. We have accordingly added the limitations in the "Conclusion" (Lines 467-473). Regarding Comment (2), we placed research questions ahead of the

“Methodology” part as suggested. In our revision, according to the structure and logic of the manuscript, we placed research objectives and questions at the end of the “Literature Review” (Lines 93-99). The changes to these parts are also highlighted in blue in the revised version.

Additional changes:

We have made substantial changes to this manuscript. The changes to the manuscript are highlighted in blue in the revised version.

1) Figure captions

According to the journal requirements, we added a separate caption for each figure in the corresponding position in the revised manuscript (Lines 167-168, 203-204, 246-247, 275, 327-328, 348, 452).

2) Supplement of relevant literature works

In this revision, we supplement six pieces of literature related to translation and interpreting teachers in the “Literature Review” part (References Entries No. 8, 9, 14, 20, 21, 23).

Both authors have read and approved the revised manuscript. We hope that our resubmission is now suitable for publication in *PLOS ONE* and we look forward to hearing from you.

Most sincerely yours,

Yi Liu and Jianyu Liu
