# Supplementary material for: Translation and interpreting teachers’ perceptions of dilemma and needs in their professional development
Source: PLoS One. 2024 Aug 9;19(8):e0289269. doi: 10.1371/journal.pone.0289269 (PMC11315291; doi:10.1371/journal.pone.0289269)
Supplement: Attachment — Submitted filename: Responses to reviewers and editors.pdf [file pone.0289269.s004.pdf]

Yi Liu and Jianyu Liu
Liaoning University
Shenyang, P. R. China
liuyi@lnu.edu.cn, ljy_0923@163.com

9 Jan, 2024

Dear Editors and Reviewers:

Thank you for your letter and for the reviewers' comments concerning our manuscript entitled "Translation and interpreting teachers' perceptions of dilemma and needs in their professional development" (ID: PONE-D-23-05313R1). Those comments are all valuable and very helpful for revising and improving our paper, as well as the important guiding significance to our researches. We have studied comments carefully and have made correction which we hope meet with approval. Revised portion are marked in blue in the paper. The main corrections in the paper and the responds to the reviewer's comments are as flowing:

Responds to the reviewer's comments:

Reviewer #1:
All comments have been addressed properly. The revised version is well-written and eligible to publish.

Response to reviewer #1:
Thank you so much for taking the time to review this manuscript.

Reviewer #2:
(No Response)

Reviewer #3:
1. Apart from research gaps, what are the main facts happening in the field currently? This has not been specifically stated in the introduction.
2. In the discussion section, there is no confirmation of the novelty of the research. This needs to be in the form of a special paragraph.
3. The results of quantitative data processing are only descriptive. Are there no other tests as supporting data for a finding in the quantitative aspect?

Response to reviewer #3:

Thank you very much for your constructive suggestions. Responses to your suggestions are as following:

1. As stated in the Introduction part, Master of Translation and Interpreting (MTI) as a newly established professional post-graduate program, most MTI teachers were shifted from the academic postgraduate programs of linguistics, applied linguistics, or literature and did not have sufficient translation or interpreting practice and professional expertise essential to the practice-oriented post-graduate program. And this insufficiency brings criticism on the professional development of MTI teachers which are unanimously perceived as the primary guarantee and core driving force for sustainable development of MTI education. Therefore, it is of practical significance to understand the difficulties and needs of MTI teachers in their professional development from their current situation.
2. At the end of the discussion section, based on the results of quantitative data analysis and the supplementation of qualitative interviews, this study proposes a three-in-one model for the professional development of MTI teachers, which is the innovation of this study (see Figure 9 and Lines 462-476).
3. Thank you for your questions and suggestions. Since the objectives of this research is to understand the current situation of difficulties and needs in the professional development of MTI teachers, this study only conducted descriptive analysis on the relevant data, and interpreted the relevant findings in combination with in-depth interviews with 7 teachers. Other quantitative statistical analysis methods (such as t-test, variance analysis, regression analysis, etc.) were not used. However, in the future, we will further explore other issues in this field and enrich the use of statistical methods in research.

Thank you again for your good comments!

Reviewer #4:

The work presents significant improvements, making it easier to understand the study's objectives as well as its limitations, but it still needs further refinement.

1. Since the analysis of responses is too simplistic, only presenting percentages and averages, it is crucial for the work to be well-grounded in theoretical terms. In this regard, in the literature review, it is not sufficient to merely identify the conducted studies; it is also necessary to present the main conclusions of recent studies within the same scope as this work,

and also highlighting the relevance and added value of this study.

2. In the final part of the literature review, when discussing the studies conducted by Liu & Xu and Liu & Zhang, the authors focus on the methodologies used in these studies rather than their conclusions and/or limitations. The text related to methodology should be placed under the subtopic "Methodology."
3. Questionnaire: Although reference is made to a previous study, here it is important to specify the type of response (5-point Likert scale: 1- ..., 2- ..., 3-..., 4- ..., and 5- ...).
4. The designation of the figures, e.g., "Fig 2. Descriptive statistics of MTI teachers' recognition of teaching problems (Mean=3.61)" does not present descriptive statistics (not plural), only one descriptive statistic – the mean. And the mean=3.61 does not make sense to be here, as it is a general average.
5. Mean: The mean is not the best measure to represent this data. For example, if you calculate the mean of the data presented in the pie charts, you may see that a lot of information is lost. The pie chart allows for a richer and more accurate analysis. Since there are many variables, I understand that you may have opted for bar charts. If making charts is not possible, you can present the values in a table with percentages per question. Suggestion: Instead of bar charts, consider using box-plots.
6. Bar charts: In the initial ones, the vertical axis displays values 0, 1, 2, 3, 4, and 5. However, the responses vary between 1 and 5. The final charts already range from 1 to 5. The vertical axis (from 1 to 5) does not refer to the mean but rather to the values of the Likert scale. The height of the bars pertains to the mean, but as mentioned earlier, it is not the best measure in this case. A legend is also necessary, as question Q1 differs from chart to chart. It is not enough to be identified in the text; a legend should appear directly on the chart to identify each question.
7. Is figure 9 your own creation, or is it based on some other work? If the latter is the case, it is necessary to give credit to the original authors.

Response to reviewer #4:

Thank you for your valuable feedback and suggestions on this research. We have carefully considered and made modifications based on your recommendations one by one. Responses to your suggestions are as following:

1. We are very sorry for our negligence of the main conclusions of recent studies. In this revised version, we have added main conclusions of recent

relevant studies as well as their limitations. We have further clarified the logic of the literature review section and proposed the research significance and concerns of this study.

2. According to the Reviewer's suggestion, we have also added key findings and limitations of these two pieces of literature to this revised manuscript.
3. Considering the Reviewer's suggestion, we have added an introduction to the scale (i.e. 5-point Likert scale) in the research instrument part (see Lines 126-129).

Suggestions 4-6:

It is really true as Reviewer suggested that the mean and the bar charts are not the best measure to represent data of this research. In the current revision, we have adjusted all the bar charts and pie charts in the manuscript to tables with labeled items, in order to more accurately present the analytical results. Please refer to Tables 3-10 for the updated representations.

7. Figure 9 "Three-in-one mechanism of MTI teachers' professional development" (Line 476) is our own creation based on the major findings of this research.

Special thanks to you for your constructive comments and suggestions!

Reviewer #5:

Relatively interesting theme and the proposed paper is original (verified by URKUND/OURIGINAL – anti plagiarism software). The work presents significant improvements, making it easier to understand the study's objectives as well as its limitations.

1. Greater attention should be paid to the identification of figures and data processing. the article must be fully revised.
2. The results of quantitative data processing are only descriptive.

Response to reviewer #5:

Thank you for your valuable suggestions on this research. Responses to your suggestions are as following:

1. Thank you once again for your constructive feedback on the data analysis and presentation of our research. We have thoroughly reanalyzed and re-presented all the data in the manuscript, and corrected any misuse of "the mean" in the original draft (please refer to Tables 3-10 for details). Your insights have been invaluable in improving the accuracy and clarity of our research.
2. Yes, the results of quantitative data processing are only descriptive. The

aim of this research is to investigate the status quo of dilemma and inner-world needs in the professional development of MTI teachers. In this study, a descriptive analysis was solely conducted on the pertinent data, with interpretations of the intensive interviews with seven MTI teachers as supplement. Other quantitative statistical analysis techniques, such as t-tests, variance analysis, regression analysis, etc., were not employed. Nevertheless, we plan to delve deeper into related topics in the future and enhance the utilization of statistical methods in our research.

Thank you again for your constructive suggestions!

Reviewer #6:

The article "Translation and interpreting teachers' perceptions of dilemma and needs in their professional development" has been well written. Authors have given sufficient background to the study. The methods used are appropriate to the title of the study. The findings provided have addressed the research questions under study.

Response to reviewer #6:

Thank you so much for taking the time to review this manuscript.

We tried our best to improve the manuscript and made some changes in the manuscript. These changes will not influence the content and framework of the paper.

We appreciate for Editors/Reviewers' warm work earnestly, and hope that the correction will meet with approval.

Once again, thank you very much for your comments and suggestions.

Both authors have read and approved the revised manuscript. We hope that our resubmission is now suitable for publication in PLOS ONE and we look forward to hearing from you.

Most sincerely yours,

Yi Liu and Jianyu Liu
